# Using an audit tool (MAPS Global) to assess the characteristics of the physical environment related to walking for transport in youth: reliability of Belgian data

**DOI:** 10.1186/s12942-016-0069-1

**Published:** 2016-11-15

**Authors:** Griet Vanwolleghem, Ariane Ghekiere, Greet Cardon, Ilse De Bourdeaudhuij, Sara D’Haese, Carrie M. Geremia, Matthieu Lenoir, James F. Sallis, Hannah Verhoeven, Delfien Van Dyck

**Affiliations:** 1 Faculty of Medicine and Health Sciences, Department of Movement and Sport Sciences, Ghent University, Watersportlaan 2, 9000 Ghent, Belgium; 2 Faculty of Medicine and Health Sciences, Department of Public Health, Ghent University, De Pintelaan 185, 4K3, 9000 Ghent, Belgium; 3 Department of Human Biometry and Biomechanics, Faculty of Physical Education and Physical Therapy, Vrije Universiteit Brussel, Pleinlaan 2, 1050 Brussels, Belgium; 4 Fund for Scientific Research Flanders (FWO), Egmontstraat 5, 1000 Brussels, Belgium; 5 Department of Family Medicine and Public Health, University of California, San Diego, 3900 Fifth Avenue, Suite 310, San Diego, CA 92103 USA

**Keywords:** Google street view, Physical activity, Walking routes, Children, Built environment, Walking for transport, Active transport

## Abstract

**Background:**

The aim was to examine inter-rater and alternate-form reliability of the Microscale Audit of Pedestrian Streetscapes (MAPS) Global tool to assess the physical environment along likely walking routes in Belgium.

**Methods:**

For 65 children participating in the BEPAS-children study, routes between their individual homes and the nearest pre-defined destination were defined. Using MAPS Global, physical environmental characteristics of the routes were audited by 4 trained auditors (2 on-site, 2 online using Google Street View). Inter-rater reliability was studied for on-site and online ratings separately. Alternate-form reliability was examined by comparing on-site with online ratings.

**Results:**

Inter-rater reliability for on-site ratings was acceptable for 68% of items (kappa range 0.03–1.00) and for online ratings for 60% of items (kappa range −0.03 to 1.00). Acceptable alternate-form reliability was reported for 60% of items (kappa range −0.01 to 1.00/r range 0.31–1.00).

**Conclusions:**

MAPS Global can be used to assess the physical environment of potential walking routes. For areas where Google Street View imagery is widely covered and often updated, MAPS Global can be completed online.

**Electronic supplementary material:**

The online version of this article (doi:10.1186/s12942-016-0069-1) contains supplementary material, which is available to authorized users.

## Background

Although engaging in active transport (walking and cycling) provides numerous health benefits, a substantial number of children and adolescents use passive transport modes (i.e. car) even when they live within feasible distances to use active transport modes to destinations [1, 2]. Hence, the promotion of active transport among youth has become an important component of efforts to increase physical activity. To effectively promote active transport in youth, it is necessary to understand its correlates [3, 4] According to ecological models [5], correlates can be identified at multiple levels (individual, social and physical environment, policy), with different correlates for each domain of physical activity. Specifically, the role of the built environment is especially important when examining correlates of youth’s active transport [1, 6, 7] Before physical environmental correlates of youth’s active transport can be adequately studied, accurate assessments of the physical environment are needed [8].

To assess macro-environmental factors of the physical neighborhood environment (e.g., land use mix, structure of buildings), self-reported questionnaires (subjective assessment) and Geographic Information Systems (GIS) (objective assessment) have mainly been used [1, 6, 7]. Observational audits have been used as an objective assessment method to provide more detailed information on micro-environmental factors (e.g., presence of speed bumps, quality of sidewalk and cycle facilities, characteristics of crossings, maintenance of buildings, presence of litter) that are hypothesized as relevant to active transport behaviors [9–11].

Completing audits on-site is resource- and time-intensive since researchers have to travel to each location to observe the environment [12, 13]. Observers are sometimes exposed to personal safety risks. To overcome these limitations of on-site audit tools, several Google Street View-based audits have been developed [12, 14–20]. With Google Street View-based audits, auditors can ‘virtually’ walk the streets and observe the physical environment. Completing a Google Street View-based audit is faster compared to on-site assessments, mainly due to travel time differences [12–15, 21]. Recent studies have shown good intra- and inter-rater reliability of Google Street View-based audit tools [16, 18–20] and reported good agreement between on-site and online audit tools [12, 14, 15, 18–20]. However, lower agreement between on-site and online assessments were reported for qualitative and more detailed data (e.g. sidewalk condition, aesthetics, physical disorder [litter]) or rapidly changing items (e.g. traffic volume) [12, 14, 15, 19, 20].

Previous Google Street View-based audits assessed physical environmental characteristics of individual street segments and not along participants’ likely walking routes. A limitation of the segment method is that it is not clear what proportion of segments needs to be observed to adequately represent a neighborhood. The route method may be better suited to assess correlates of active transport, especially among children and adolescents [22, 23]. Physical environmental factors along walking routes (e.g. presence of driveways along the sidewalk, obstructions on the sidewalk, dangerous crossings) were previously identified as being important for transport mode decisions among youth [23]. Most Google Street View-based audits have been evaluated in U.S. environments [14–17]. However, physical environmental correlates and active transport behaviors are likely to differ between continents and countries, so there is a need to develop and assess audit tools which can be used in other countries and continents than the U.S.

The Microscale Audit of Pedestrian Streetscapes (MAPS) Global tool has recently been developed to assess the physical environment along walking routes using a uniform method that allows comparisons across countries and continents. MAPS Global was based on an instrument developed and evaluated in the US [24, 25] but incorporated items from environmental audits and questionnaires developed for several continents. The goal was to create an instrument that is suitable for the widely varying streetscape features around the world but also allows international comparability. An additional aim of the MAPS Global tool is to be able to use it either on-site or online with Google Street View. In that way, auditors can choose how to complete the audit tool depending on resources and availability of Google Street View imagery. High-resolution Google Street View imagery is not available for all areas and countries (e.g. remote areas, countries in Africa and the Middle East). Prior to conducting cross-country comparisons, it is necessary to investigate the reliability of the Maps Global tool in diverse international environments.

The present study examined the reliability of the MAPS Global tool in a Flemish (Belgian) context. Flanders (northern part of Belgium) is characterized by a mild climate and a flat landscape in which higher active transport among youth has been reported compared to other countries (e.g. US, Australia, Spain) [26]. The first aim of the present study was to investigate the inter-rater reliability of the MAPS Global tool in Belgium. Inter-rater reliability was studied for on-site ratings as well as for online ratings (Google Street View). The second aim was to examine the alternate-form reliability of the MAPS Global tool by comparing on-site ratings with online ratings (Google Street View).

## Methods

### Procedure

#### MAPS Global tool

The MAPS Global tool was developed to assess macro- and micro-environmental factors of routes relevant to walking and cycling, by trained observers conducting observations either on-site or using Google Street View. The MAPS Global tool can be found online [27]. MAPS Global was created by a team led by researchers of the University of California San Diego, in collaboration with investigators of the IPEN (International Physical activity and Environment Network) Adolescent Study [28]. The tool includes micro-scale environmental characteristics about streets, sidewalks, intersections and design characteristics. The MAPS Global tool was an adapted version of the original MAPS [24] which was primarily based on the Audit Tool Analytic Version [29]. Inter-rater reliability of the original MAPS was found to be high [24]. The difference between the original MAPS and MAPS Global is that the latter was designed for international use. MAPS Global drew items and concepts from audit tools and self-reported questionnaires developed for the US, Australia, Europe and Asia (MAPS [24], Bikeability Toolkit [30], SPACES [31], ALPHA [32], REAT [33], FASTVIEW [18], school audit tool used in SPEEDY/ISCOLE study [34], EAST_HK [35]). Draft versions were reviewed by numerous investigators, and MAPS Global is currently being evaluated in 5 countries.

MAPS Global consists of four main sections: (1) route, (2) segment, (3) crossing, (4) cul-de-sac. The route section includes items to assess the overall experience of the routes. This section consists of three subscales including Land use/destinations (e.g., types of residential use, number of fast food restaurants), Streetscape features [e.g., number of public transit stops, presence of street amenities (trash bins)], and Aesthetics and Social environment (e.g., presence of hardscape features (fountains, sculptures, art), presence of anyone walking). For the route section, the items were audited for both sides of the street. In the segment section, items to assess more detailed features for each segment of the route were included, such as characteristics of the sidewalk (e.g., width, buffer, trees or other coverage of the sidewalk), slope, building setbacks (distance from sidewalk to buildings), building heights, characteristics of buildings (e.g. number of driveways) and bicycle facilities (e.g., type of bicycle lane or protected path). For the segment section, only one side of the street was audited. The first segment was audited on the side of the street where the child’s home was located. A route could consist of multiple segments and crossings. The crossing section included presence of crossing signals, pedestrian protection (e.g., curbs, protected refuge islands), types of crosswalk treatment (e.g., marked crosswalk), visibility at corners, width of crossings and bicycle amenities (e.g., bike box). Audits were conducted only for the portion of the intersection the observer crossed over, except for the item regarding intersection control where the whole intersection (e.g., all stop signs of the different crossing legs) was audited. The cul-de-sac section included items about the proximity of the cul-de-sac opening from the participant’s home, amenities at the cul-de-sac (e.g., basketball hoops) and visibility of cul-de-sac area from the participant’s home. This section was only completed if the child’s residence was situated within 120 m of a cul-de-sac.

The proposed subscales are generally consistent with the scales of the MAPS tool [24]. An overview of the subscales for each section can be found in Table 1 and Table 2.

**Table 1:** Summary results of the inter-rater reliability of MAPS Global: overview per (sub)section and subscale

	Number of items	Inter-rater reliability between on-site ratings			Inter-rater reliability between online ratings		
		ICC/kappa range	Moderate-to-perfect agreement n (%)	Fair/poor agreement n (%)	ICC/kappa range n (%)	Moderate-to-perfect agreement n (%)	Fair/poor agreement n (%)
All	119	0.03–1.00	81 (67.5)	10 (8.3)	−0.03–1.00	72 (60.0)	21 (17.5)
ROUTE (n = 65)	61	0.03–1.00	41 (67.2)	5 (8.2)	−0.03–0.97	33 (54.1)	12 (19.7)
Land use/destinations	31	0.03–1.00	22 (71.0)	2 (6.5)	−0.03–0.97	19 (61.3)	6 (19.4)
Residential density	4	0.66–0.80	4 (100)	–	0.47–0.78	4 (100)	–
Shops	8	0.92–1.00	5 (62.5)	–	0.51–0.97	5 (62.5)	–
Restaurant and entertainment	4	0.03–0.93	2 (50.0)	2 (50.0)	−0.03–0.93	2 (50.0)	2 (50.0)
Institutional/Services	3	0.90–0.98	3 (100)	–	0.30–0.93	2 (66.7)	1 (33.3)
Public recreation	4	0.66–0.80	3 (75.0)	–	0.33–0.59	2 (50.0)	1 (25.0)
private recreation	2	0.80	1 (50.0)	–	0.32	–	1 (50.0)
Worship land uses^a^	1	0.96	1	–	0.77	1	–
School land uses^a^	1	0.85	1	–	0.79	1	–
Pedestrian zone land uses^a^	1	0.47	1	–	0.64	1	–
Age restricted land uses^a^	1	1.00	1	–	0.66	1	–
Liquor related land uses^a^	1	N/A	–	–	0.58	1	–
Industrial land uses^a^	1	N/A	–	–	0.33	–	1
Streetscape	19	0.48–0.98	11 (57.9)	–	0.42–0.82	9 (47.4)	–
Aesthetics and Social	11	0.13–0.91	8 (72.7)	3 (27.3)	−0.02–0.87	5 (45.5)	6 (54.5)
Segment (n = 220)	29	0.23–0.97	26 (89.7)	2 (6.9)	−0.01–0.95	21 (72.4)	7 (24.1)
Setback and building height	4^b^	0.71–0.83	4 (100)	–	0.48–0.67	4 (100)	–
Building height to road width ratio	5	0.71–0.83	5 (100)	–	0.48–0.89	5 (100)	–
Sidewalk	8	0.23–0.97	6 (75.0)	2 (25.0)	−0.01–0.67	5 (62.5)	3 (37.5)
Buffer	2	0.69–0.89	2 (100)	–	0.45–0.55	2 (100)	–
Bike infrastructure	3	0.51–0.89	3 (100)	–	0.38–0.95	2 (66.7)	1 (33.3)
Building surveillance^a^	1	0.48	1	–	0.81	1	–
Shade	3	0.61–0.87	3 (100)	–	0.55–0.67	3 (100)	–
Pedestrian connectivity	3	0.85	2 (66.7)	–	0.30–0.33	–	2 (66.7)
Informal path^a^	1	0.77	1	–	0.84	1	–
Hawkers/Shops^a^	1	0.95	1	–	0.04	–	1
High (car) street lights^a^	1	0.61	1	–	0.55	1	–
Low (pedestrian) street lights^a^	1	0.75	1	–	0.65	1	–
Crossing (n = 124)	23	−0.01 to 1.00	12 (50.0)	3 (12.5)	0.27–0.95	16 (66.6)	2 (8.3)
Crosswalk amenities	7	0.34–1.00	2 (28.6)	1 (14.3)	0.38–0.94	4 (57.1)	1 (14.3)
Curb quality/presence	3	0.71–0.88	3 (100)	–	0.69–0.94	3 (100)	–
Intersection control and signage	7	−0.01–1.00	4 (57.1)	1 (14.3)	0.66–1.00	6 (85.7)	–
Bike	3	0.80–0.90	2 (66.7)	–	0.66–0.76	2 (66.7)	–
Overpass^a^	1	N/A	–	–	N/A	–	–
Road width^a^	1	0.90	1	–	0.78	1	–
Visibility^a^	1	0.38	–	1	0.27	–	1
CUL-DE-SAC (n = 6)	6	0.67–0.76	2 (33.3)	–	0.76–1.00	2 (33.3)	–

Some items no kappa or ICC could be calculated as at least one variable was constant

*ICC* intraclass correlation coefficient

^a^ Single item; ^b^ 4 items are also included in subscale Building Height to Road Width Ratio

**Table 2:** Summary results of the alternate-form reliability of MAPS Global: overview per (sub)section and subscale

	Number of items	Alternate-form reliability (on-site–online)			
		ICC/kappa range	r range	Moderate-to-perfect agreement n (%)	Fair/poor agreement n (%)
All	119	−0.01 to 1.00	0.31–1.00	72 (60.0)	17 (14.2)
Route (n = 65)	61	0.03–1.00	0.31–1.00	36 (59.0)	5 (8.2)
Land use/destinations	31	0.41–0.81	0.31–1.00	24 (77.4)	–
Residential density	4	0.41–0.81	–	4 (100)	–
Shops	8	–	0.49–0.92	5 (62.5)	–
Restaurant and entertainment	4	–	0.31–0.94	4 (100)	–
Institutional/services	3	–	0.63–0.94	3 (100)	–
Public recreation	4	–	0.32–0.84	2 (50.0)	–
Private recreation	2	–	0.70–1.00	2 (100)	–
Worship land uses^a^	1	–	0.78	1	–
School land uses^a^	1	–	0.81	1	–
Pedestrian zone land uses^a^	1	–	0.48	1	–
Age restricted land uses^a^	1	–	0.70	1	–
Liquor related land uses^a^	1	–	N/A	–	–
Industrial land uses^a^	1	–	N/A	–	–
Streetscape	19	0.54–0.91	0.32–0.87	9 (47.4)	–
Aesthetics and social	11	−0.03–0.74	–	3 (27.3)	8 (72.7)
Segment (n = 220)	29	−0.01–0.86	0.81	23 (79.3)	6 (20.7)
Setback and building height	4^b^	0.48–0.61	–	4 (100)	–
Building height to road width ratio	5	0.48–0.61	0.81	5 (100)	–
Sidewalk	8	−0.01–0.82	–	5 (62.5)	3 (37.5)
Buffer	2	0.62–0.66	–	2 (100)	–
Bike infrastructure	3	0.31–0.86	–	2 (66.7)	1 (33.3)
Building surveillance^a^	1	0.57	–	1	–
Shade	3	0.47–0.68	–	3 (100)	–
Pedestrian connectivity	3	0.34–0.36	–	–	2 (66.7)
Informal path^a^	1	0.58	–	1	–
Hawkers/shops^a^	1	0.78	–	1	–
High (car) street lights^a^	1	0.50	–	1	–
Low (pedestrian) street lights^a^	1	0.64	–	1	–
CROSSING (n = 124)	23	−0.01–1.00	0.79	12 (50.0)	3 (12.5)
Crosswalk amenities	7	0.19–0.96	–	2 (28.6)	1 (14.3)
Curb quality/presence	3	0.64–0.76	–	3 (100)	–
Intersection control and signage	7	−0.01–1.00	–	4 (57.1)	1 (14.3)
Bike	3	0.65–0.66	–	2 (66.7)	–
Overpass^a^	1	N/A	–	–	–
Road width^a^	1	–	0.79	1	–
Visibility^a^	1	−0.02	–	–	1
CUL-DE-SAC (n = 6)	6	0.76–1.00	–	2 (33.3)	–

Some items no kappa or ICC could be calculated as at least one variable was constant

*ICC* intraclass correlation coefficient; *r* Pearson correlation coefficient

^a^ Single item; ^b^ 4 items are also included in subscale Building Height to Road Width Ratio

##### Route selection

Data from the Belgian Environmental Physical Activity Study in children (BEPAS-child) were used to obtain socio-demographic information [age, sex, socio-economic status (SES)] of children 10–12 years and to select their home addresses for the present study. More detailed information about the selection procedure and the measurements used in the BEPAS-child study can be found elsewhere [36]. Written consent was obtained from the parents of participating children, and the study was approved by the Ethics Committee of the Ghent University Hospital. For the present study, 68 home addresses were randomly selected from diverse urban and suburban environments of varying SES levels in Flanders and were used to define routes. One route per child was defined starting at each child’s home and moving toward the nearest pre-determined destination (e.g. cluster of shops, services, park, school) along the street network. The maximum distance of each route was approximately 400 meters, which is a feasible walking distance [24]. The routes were identified using Google Earth (Microsoft Windows, 2013 Google Inc.) and printed on maps to guide auditors through the exact walking routes.

Routes consisted of several street segments and crossings. Segments were defined as the part of the street between two crossings, between an intersection and cul-de-sac, or if the name of a street changed. Using street segments to assess the physical environment was based on the methodology of previously developed audit tools (MAPS tool [24], Audit Tool Checklist Version [29], Pikora-SPACES instrument [31] and Irvine-Minnesota Inventory [37]). The crossings section of MAPS Global was completed when the auditor crossed the street, whether a marked pedestrian crossing existed or not. Cul-de-sacs sections were completed when the dead-end part of the child’s street was within 120 meters of the child’s home. For each route, detailed information (i.e., number of segments, crossings, cul-de-sacs with start- and endpoints) was recorded in a Microsoft Access database, developed by researchers at the University of California, San Diego. Of the 68 selected routes, 3 were excluded due to almost complete overlap with other routes. In total, 65 routes including 220 segments, 124 crossings and 6 cul-de-sacs were audited by four auditors (2 auditors on-site, 2 auditors using Google Earth/Google Street View).

##### Training procedure

Prior to auditing the pre-defined routes, all auditors were trained by a Belgian data manager. This data manager had viewed a training webinar and was certified by researchers from the University of California, San Diego, who developed training materials and procedures. The standard one-day training provided by the data manager included specific instructions and definitions, with most items illustrated with photographs. Training included the use of the MAPS Global tool in the field where all auditors could raise questions. After training, a certification period was required in which 5 diverse routes (i.e., no routes that were part of the study) were rated by the four auditors. For certification of auditors 95% agreement with the trainers’ scores was required.

##### Data collection procedure

Four auditors (2 on-site, 2 online) audited the neighborhood environmental characteristics of the 65 selected routes using MAPS Global. Both on-site auditors walked along each route to independently audit the environmental characteristics. Route changes were possible if they were agreed upon by both on-site auditors. For example, when on-site auditors crossed the street due to unexpected circumstances (e.g., road construction), a crossing section was completed along with a new segment section. Changes were communicated to the data manager who informed the other two auditors using Google Street View. Online auditors used the same MAPS Global tool to independently audit the environmental characteristics using Google Earth and Google Street View. To avoid bias in comparing the two observation modes, the researchers who audited the routes by on-site assessment did not audit the routes by Google Street View.

### Data analysis

The Statistical Package for the Social Sciences for Windows version 20 (SPSS Inc., Chicago, IL, USA) was used to perform statistical analyses, and tests were considered significant at *p* < 0.05. Means, standard deviations (SD) and percentages were used to describe the routes.

#### Inter-rater reliability

To assess inter-rater reliability, audits were compared between (1) the two on-site auditors and (2) the two online auditors at the individual item-level by using the kappa test for agreement of categorical variables (n = 90) and intraclass correlation coefficient (ICC) for continuous items (n = 29). To interpret the kappa and ICC results, ratings by Landis and Koch [38] were used: 0.00–0.20 (poor), 0.21–0.40 (fair), 0.41–0.60 (moderate), 0.61–0.80 (substantial), 0.81–1.00 (almost perfect). For the kappa statistics, ratings with negative kappas between −0.10 and 0 were interpreted as no agreement since a negative kappa represents agreement worse than expected or no agreement. If at least one variable was constant, indicating no variance in responses of one or both auditors, no kappa’s could be calculated. Percentage agreement was calculated for all items to determine the proportion of occasions that auditors gave the same score. Percentage agreement above 70% was considered high [39].

#### Alternate-form reliability

Alternate-form reliability is the reliability between two different methods (here: on-site and online) on the same “outcome” (here: route characteristic) [40]. To compare the online and on-site audits, one pair of an on-site and an online auditor was randomly selected, of which the results were presented. Preliminary analyses indicated very similar results in reliability when analysing the data from other combinations of auditors.

Kappa statistics and Pearson correlations were calculated between the on-site ratings and online ratings at the individual item-level. Pearson correlations were calculated for the 29 continuous items. Correlations were considered low (≤0.30), moderate (0.31–0.50) and high (>0.50) (see [43]). Kappas were calculated for the remaining categorical items (n = 90). To interpret the kappa statistics, the ratings by Landis en Koch were used [38]. Percentage agreement was calculated for all categorical items, above 70% was considered high [39].

## Results

### Descriptive information

On average, routes consisted of 3.4 ± 1.3 segments and 1.9 ± 1.4 crossings. Only 6 routes (9.2%) had a cul-de-sac. Auditor 1 (on-site) had an average observation duration of 33.7 ± 14.4 min/route and auditor 2 (on-site) had 34.3 ± 16.7 min/route. The online ratings by auditor 3 (Google Street View) lasted 30.0 ± 13.9 min/route and 30.7 ± 11.1 min/route by auditor 4 (Google Street View). The response frequency of each individual item of MAPS Global audited by auditor 1 (on-site), auditor 2 (on-site), auditor 3 (Google Street View) and auditor 4 (Google Street View) is shown in an additional file [see Additional file 1].

### Inter-rater reliability

Table 1 presents a summary of the inter-rater reliability results for the on-site ratings and online ratings. Complete results, with percentage agreement, for the individual items are reported in an additional file [see Additional file 2]. Of the 119 individual items rated on-site, 70 items generated substantial-to-almost perfect agreement (45 items for the online ratings), 11 items moderate (27 items for the online ratings), 7 items fair (15 items for the online ratings) and 3 items poor or no agreement (6 items for the online ratings). Kappas or ICC values could not be calculated for 28 items and 26 items for the on-site and online ratings, respectively, as at least one of the items had no variance in responses. Of the 10 on-site and 21 online items with fair to poor inter-rater reliability, most were observed in the route section (n on-site = 5; n online = 12), and of these most items were in the Aesthetics and Social subscale. Most of the lower reliability scores in the segment section (n onsite = 2; n online = 7) were in the Sidewalk subscale. There were just a few low reliability scores in the crossing section (n on-site = 3; n online = 2).

#### Route

Of the 61 individual items in the route section, 41 on-site ratings and 33 online ratings had moderate-to-almost-perfect inter-rater reliability.

When examining the results by subscale, inter-rater reliability was moderate-to- almost-perfect for the majority of items of the Land use/destinations subscale (n on-site = 22 of the 31 items, n online = 19 of the 31 items). For the on-site ratings, two items in the subscale Restaurant and Entertainment generated fair/poor agreement [“number of entertainment destinations along the route” (ICC = 0.21), “number of cafés or coffee shops along the route” (ICC = 0.03)]. For the online ratings, five items generated fair agreement and one item generated poor agreement [“number of entertainment destinations along the route” (ICC = −0.03)].

In the Streetscape subscale, 11 items of the 19 items showed moderate-to-almost-perfect agreement for the on-site ratings and 9 of the 19 items for the online ratings. The highest reliability items dealt with transit stops along the route. In the Aesthetics and Social environment subscale, 8 of the 11 items showed moderate-to-almost-perfect agreement for the on-site ratings with the highest score for presence of natural bodies of water along the route (k = 0.91). For the online ratings, 5 of the 11 items showed moderate-to-almost-perfect agreement with the highest score for presence of anyone walking (k = 0.87). Fair agreement for the on-site ratings was found for the items maintenance of buildings along the route (k = 0.32) and presence of graffiti along the route (k = 0.27), and poor agreement for maintenance of landscaping along the route (k = 0.13). For the online ratings, 6 items generated fair, poor or no agreement: presence of hardscape features (k = 0.23), presence of softscape features (k = 0.36), presence of noticeable/excessive litter along the route (k = 0.28), maintenance of landscaping along the route (k = 0.04), maintenance of buildings along the route (k = 0.00), presence of noticeable/excessive dog fouling along the route (k = −0.02).

#### Segment

At the segment section, most items generated moderate-to-almost-perfect agreement for both the on-site ratings (26 of the 29 items) and the online ratings (21 of the 29 items). For the online ratings, the majority of the fair or poor items were in the Sidewalk subscale. Five items generated fair agreement in online ratings: width of the majority of the sidewalk (k = 0.37), presence of cars blocking the sidewalk or pedestrian street/zone (k = 0.32), presence of mid-segment crossings along the segment (k = 0.33), presence of a pedestrian bridge/overpass/tunnel at mid-segment crossing along the segment (k = 0.30), presence of signs or sharrows indicating bicycle use (k = 0.38). Poor or no agreement was only reported for the online ratings for slope of the segment (k = −0.01) and presence of hawkers or shops on the sidewalk (k = 0.04).

#### Crossing

Twelve and 16 of the 23 items showed substantial-to-perfect agreement for the on-site ratings and online ratings, respectively. For the on-site ratings, two items in the subscales Crosswalk Amenities [“crosswalk of crossing in different material than road” (k = 0.34)] and Visibility [“poor visibility at the corners of the crossing” (k = 0.38)] generated fair agreement. No agreement was reported for one item in the subscale Intersection Control and Signage [“presence of stop signs at intersection” (k = −0.01)]. For online ratings, two items in the subscales Crosswalk Amenities [“presence of raised crosswalk (k = 0.38)] and Visibility [“poor visibility at the corners of the crossing” (k = 0.27)] showed fair agreement. No items generated poor or no agreement.

#### Cul-de-sac

In the cul-de-sac section, 2 of the 6 items showed substantial agreement for the on-site and online ratings [proximity of opening cul-de-sac to participant’s home (k = 0.76), visibility of cul-de-sac from the participant’s home (k = 0.67)]. For the remaining items, kappas could not be calculated due to absence of the features.

### Alternate-form reliability

Table 2 reports results of the alternate-form reliability analyses. Complete results with percentage agreement for the individual items were reported in an additional file [see Additional file 2] that also presents usability of the MAPS Global tool in Belgium of all the individual items.

Of the 119 individual items, 49 items showed substantial-to-almost-perfect agreement and 23 items moderate agreement. Fair, poor or no agreement was found for 17 items. No kappas or correlations could be calculated for 30 items. Of all the fair and low scores, 8 items were part of the route section with the majority in the subscale Aesthetics and Social. Six fair and poor items were part of the segment section and most items were in the Sidewalk subscale. Three poor items were part of the crossing section.

#### Route

Of the 61 individual items in the route section, 36 showed moderate-to-almost-perfect agreement. For the subscale Land use/destinations on the route, the majority of the items (24 of the 31 items) showed moderate-to-almost-perfect agreement.

In the Streetscape subscale, 9 items (of the 19 items) showed moderate-to-almost-perfect agreement with highest scores for availability of tram/streetcar at transit stops along the route (k = 0.91) and number of public transit stops along the route (r = 0.87).

In the Aesthetics and Social environment subscale, 8 of the 11 items showed fair, poor or no agreement with lowest scores for presence of dog fouling (k = −0.03) and maintenance of landscaping (k = 0.17). None of the items showed almost-perfect agreement.

#### Segment

The majority of the items on the segment Sect. (23 of the 29 items) showed moderate-to-almost-perfect agreement, with the highest score reported for type of bicycle lane of the segment (k = 0.86). Six items generated fair, poor or no agreement, with most on the Sidewalk subscale; i.e., width of the sidewalk (k = 0.38), slope of the segment (k = −0.01), percentage of properties protected by gates, walls or tall fences (k = 0.22), mid-segment crossing (k = 0.36), pedestrian bridge/overpass/tunnel at mid-segment crossing (k = 0.34) and signs or sharrows indicating bicycle use (k = 0.31).

#### Crossing

Moderate-to-perfect agreement was found for about half the items in the crossing section (12 of the 23 items). Three items showing poor agreement were on the Crosswalk Amenities scale (“crosswalk in different material than road” (k = 0.19), Intersection Control and Signage [“presence of stop signs at intersection” (k = −0.01)] and Visibility [“poor visibility at the corners of the crossing” (k = −0.02)].

#### Cul-de-sac

For the cul-de-sac section, 2 of the 6 items showed substantial-to-perfect agreement [“proximity of opening cul-de-sac to participant’s home (k = 0.76), visibility of cul-de-sac from participant’s home (k = 1.00)].

## Discussion

This study evaluated the inter-rater reliability and alternate-form reliability of the MAPS Global audit tool to assess the physical environment along potential walking routes in Belgium. Overall, 68% of all items on MAPS Global demonstrated moderate-to-high inter-rater reliability for the on-site assessments. Inter-rater reliability for the Google Street View assessments was at least acceptable for 60% of items. Acceptable or better alternate-form reliability between the on-site and the Google Street View assessments was reported for 60% of items. Results consistently indicated a somewhat higher inter-rater reliability for audits completed on-site compared to online. However, inter-rater reliability results were generally high in both assessment-methods and were higher than observed in some other studies [18, 20, 41]. Only a study of Kelly and colleagues [16] reported higher inter-rater results for Google Street View ratings of the physical neighborhood environment, in which 95% of the items generated substantial to perfect agreement.

In previous studies, low agreement between raters was found, both on-site and with Google Street View, for items on quality and aesthetics due likely to the subjectivity required by the items. For example, Gullón and colleagues [20] found low agreement between on-site raters, but also between Google Street View raters, for walking and cycling surface (e.g., path smoothness, path material), aesthetics (e.g., maintenance of gardens, attractiveness, cleanliness) and traffic controls in the neighborhood environment when evaluating the M-SPACES in Spain. In the present study, most low-reliability items were observed among items with little variance in the answers, as the percentage agreement was generally high for those items (>70% percentage agreement) [42, 43]. The few remaining items with low inter-rater reliability were part of the Aesthetics and Social environment subscale [“maintenance of buildings along the route” (for on-site and online ratings), “maintenance of landscaping along the route” (for online ratings), “presence of noticeable/excessive litter” (for online ratings)] and the Sidewalk subscale [“width of the sidewalk” (for online ratings)]. Some of the items are inherently subjective, such as “excessive litter” and “maintenance” of buildings and landscaping. However, some features may be particularly difficult to see online due to insufficient resolution of the photographs or obstructed views from traffic or parked cars.

The alternate-form reliability results in the present study were similar to previous studies comparing on-site and Google Street View assessments of the neighborhood environment [12, 14, 15, 17, 18, 20]. These studies all reported acceptable scores between the on-site and online ratings for the majority of the items (ranging from 52 to 83% of the items), which is in line with present results (i.e. 60% of the individual items generated moderate to almost perfect agreement). Only one study evaluated a Google Street View-based audit (EGA-Cycling) focusing on the physical environment along routes [41]. The present study showed higher scores compared to the results found in the EGA-Cycling study. However, EGA-Cycling consisted of more detailed cycling-related items, which tended to produce low scores, possibly because the features were difficult to see on the photographs.

In the MAPS Global audit tool, 11 of the 17 low alternate-form reliability item scores showed high percentage agreement (>70%), indicating low variance in the items [42, 43]. Therefore, present results should not be taken as evidence of poor alternate-form reliability. Because MAPS Global was designed to be globally applicable, it is expected that some items will have low frequency of occurrence in each country. But it is important to include items that are common in some countries and rare in others. Of the 6 remaining items with low percentage agreement, most items (“presence of softscape features”, “maintenance of buildings”, “maintenance of landscaping”, “extent of graffiti, litter and dog fouling”) were part of the route section and were in the Aesthetics and Social subscale. The low agreement across observation modes is further evidence that online observations are not well suited for items that involve judgments of quality, aesthetics and changeable items, and other authors have come to similar conclusions [14, 15, 18, 20, 41]. Another possible explanation for these low-scored items could be that these items needed observation along the entire route which makes it difficult for the auditors to provide an overall impression of for example the extent of graffiti, litter or dog fouling along the route. The perspective of Google Street View images, from a car driving down the road, does not always allow auditors to observe detailed environmental features and features from a pedestrian view. This limitation of Google Street View may also explain the low scores of the items regarding the maintenance of buildings/landscaping and the extent of graffiti, litter or dog fouling along the route which require more detailed observation. The remaining low scored items (“width of the sidewalk”, “visibility at the corners of a crossing”) require observation from a pedestrian view which Street View does not provide.

Based on present results, most items of the MAPS Global tool can be observed on-site as well as by Google Street View in Belgium. For countries and areas that are widely covered by Google Street View imagery and where the imagery is often updated (like in Belgium), most of the MAPS Global tool can be completed reliably online. Completing the audit through Google Street View is advisable due to lower time and financial costs of travel. When researchers prefer to complete the audits on-site, routes should be carefully planned based on their location, to minimize time needed to travel between the routes. In the present study, travel times by bicycle to the starting point ranged from 1 to 45 min, and on-site raters were able to complete 6–8 routes per day. Additionally, Google Street View is available at any moment, and auditors are not restricted due to adverse weather conditions or concerns about personal safety. However, online observation sacrifices the ability to collect high-quality aesthetic items or other items that required detailed observations such as sidewalk width. For areas and countries where Google Street View imagery is not available (e.g., remote areas) or is not very often updated, MAPS Global can be completed on-site. Another benefit of Street View auditing is that the same raters could work from a central location and use the same quality control methods to observe routes any place in the world with adequate Street View data. This approach could enhance quality and comparability of observations. We also argue that if environmental assessments are the main outcome of the study interest, on-site assessments are preferred over online rating, but if environmental assessments are part of a larger scale project in which environmental characteristics are one aspect of the study, Google Street View can also be a very good research tool. It would be useful to explore improvements to the two items and response options of “maintenance of buildings along the route” and “maintenance of landscaping along the route” to enhance their reliability. Perhaps changing response options from percentages to yes/no or many/few/little/none would increase reliability for those two items. For many items a constant response was given by all auditors, usually indicating absence of the feature. A full list of low-frequency items in this Belgian sample of routes is provided in Additional file 2. Instead of removing items that are rare or nonexistent from the MAPS Global tool, it is important to retain those items for the purpose of allowing comparability across different countries since the MAPS Global tool is designed for international use.

The present study has important strengths. First, assessments were conducted on overall routes, but also on segments and crossings across different environments. Second, reliability analyses were conducted on a large set of environmental characteristics (i.e. macro- and micro-environmental factors). To ensure adequate variability, audits were conducted in heterogeneous neighbourhoods, which were selected to vary in residential density, street connectivity, socio-economic status, vegetation density, and mixed-use given the Belgian situation. The present study has some limitations. First, the reliability of the MAPS Global tool has been tested only within Belgium, which is characterized by a flat landscape and mild climate, a well-developed walking and cycling infrastructure etc. This may limit generalization of the findings to its use in other countries and studies. However, the routes were selected to maximize geographic and socio-economic variation within the study area. Another limitation involved the small number of auditors who completed the MAPS Global tool, which may affect the generalizability of the results. However, the selection of four auditors and comparing ratings between two auditors was based on the methodology of similar studies testing reliability of audit tools to assess the physical environment [16–18, 44]. Third, only one walking route per child to the nearest pre-defined destination was defined by a team of researchers. Those routes may differ from the youth’s actual routes to different destinations. Future research could use GPS devices to track in detail youth’s actual routes to different destinations. Finally, the number of routes was small, but the sample size of routes is sufficient for assessing reliability.

## Conclusions

The MAPS Global tool generated high reliability for the majority of items in this Belgian study, supporting its use in similar settings. MAPS Global can be used in studies to assess characteristics of the physical environment along walking routes, either by conducting the audit on-site or online by Google Street View. Once its reliability is confirmed in other countries, the MAPS Global tool can be completed with Google Street View for countries and areas that are widely covered by Google Street View imagery and where the imagery is often updated. Using MAPS Global online is not recommended for some detailed features related to aesthetics or for features requiring observation from a pedestrian view, such as sidewalk width.

## Additional files

**Additional file 1. **Response frequencies of MAPS Global tool. This file provides the response frequency of each individual item of the MAPS Global tool audited by on-site ratings of auditor 1, auditor 2, and online ratings of auditor 3 and auditor 4 (Google Street View).

**Additional file 2. ** Inter-rater reliability, alternate-form reliability and usability of MAPS Global tool in Belgium. This file provides the results regarding inter-rater reliability, alternate-form reliability and usability of the MAPS Global tool in Belgium to assess the physical environment related with walking for transport in youth.

## Abbreviations

MAPS: microscale audit of pedestrian streetscapes
GIS: geographic information systems
US: United States
SES: socio-economic statusl
ICC: intraclass correlation coefficient
