# Supplementary material for: Using an audit tool (MAPS Global) to assess the characteristics of the physical environment related to walking for transport in youth: reliability of Belgian data
Source: Int J Health Geogr. 2016 Nov 15;15:41. doi: 10.1186/s12942-016-0069-1 (PMC5111220; doi:10.1186/s12942-016-0069-1)
Supplement: Supplementary file 2 — Additional file 2. Inter-rater reliability, alternate-form reliability and usability of MAPS Global tool in Belgium. This file provides the results regarding inter-rater reliability, alternate-form reliability and usability of the MAPS Global tool in Belgium to assess the physical environment related with walking for transport in youth. [file 12942_2016_69_MOESM2_ESM.docx]

| **Additional file 2. Inter-rater reliability, alternate-form reliability and usability of MAPS Global tool in Belgium** | | | | | | | | | | |  |
| --- | --- | --- | --- | --- | --- | --- | --- | --- | --- | --- | --- |
|  |  | **Inter-rater reliability between on-site ratings** | | | **Inter-rater reliability between online ratings** | | | **Alternate-form reliability**  **(on-site – online)** | | | **Usability in Belgium** |
| Item | Response options | ICC | Kappa | % agreement | ICC | Kappa | % agreement | r | Kappa | % agreement |  |
| ***ROUTE (n=65)*** | | | | | | | | | | |  |
| ***Land use/destinations*** | | | | | | | | | | |  |
| 1) What type of residential uses?   - Single family houses - Multi-unit homes (duplex, 4-plex row house) - Apartments or condominiums - Apartments above street retail | No/Yes  No/Yes  No/Yes  No/Yes |  | 0.72  0.80  0.71  0.66 | 86.2  93.8  86.2  83.1 |  | 0.78  0.53  0.47  0.58 | 89.2  86.2  73.8  81.5 |  | 0.81  0.77  0.62  0.41 | 90.8  92.3  81.5  73.8 | V  V  V  V |
| 2) How many of the following types of non-residential destinations are present?  a. Fast food restaurant (national or local chain, primarily sells burgers, chicken, pizza, etc.)  b. Sit-down restaurant or bar (all-ages)  c. Grocery/supermarket  d. Convenience store (may also be a gas station)  e. Café or coffee shop  f. Bakery  g. Age-restricted bar/nightclub  h. Liquor or alcohol store  i. Bank or credit union  j. Drugstore/pharmacy  k. Health-related professional (e.g. chiropractor, Dr. office, private health care facilities)  l. Entertainment (e.g. movie theater, arcade)  m. Other service (e.g. salon, accountant, dry cleaner)  n. Other retail (e.g. books, clothing, hardware)  o. Place of worship (e.g. church, synagogue, convent, mosque, etc.)  p. School  q. Private indoor recreation (e.g. commercial gyms, dance clubs)  r. Public indoor recreation (e.g. community center)  s. Private outdoor recreation (e.g. private golf course)  t. Public outdoor pay recreation (e.g. pool)  u. Public park  v. Trail  w. Warehouse/factory/ industrial  x. Pedestrian street or zone |  | 0.93  0.80    0.96  0.92  **0.03**  0.98  1.00  N/A^a^  0.98  1.00  0.96  **0.21**  0.90  0.95  0.96  0.85  0.80  0.80  N/A^a^  0.66  0.74  N/A^a^  N/A^a^  0.47 |  | 89.2  78.1  90.8  84.6  **89.2**  96.9  100  100  95.3  100  86.2  **93.8**  83.1  78.5  98.5  93.8  98.5  98.5  98.5  98.5  92.3  100  100  93.8 | 0.89  0.93    0.84  0.51  **0.34**  0.97  0.66  0.58  0.93  0.81  **0.30**  **-0.03**  0.83  0.89  0.77  0.79  **0.32**  N/A^a^  N/A^a^  **0.33**  0.59  N/A^a^  **0.33**  0.64 |  | 87.7  76.9  75.4  81.5  **87.7**  93.8  96.9  95.3  89.2  92.3  **73.8**  **86.2**  56.9  70.8  92.3  90.8  **93.8**  98.8  98.5  **98.5**  73.8  81.5  **83.1**  87.7 | 0.94***  0.77***  0.66***  0.49***  0.35**  0.92***  0.70***  N/A^a^  0.94***  0.86***  0.63***  0.31  0.81***  0.83***  0.78***  0.81***  0.70***  N/A^a^  1.00***  0.32  0.84***  N/A^a^  N/A^a^  0.48*** |  |  | V  V  V  V  V  V  V  V  V  V  V  V  V  V  V  V  V  V  V  V  V  -  V  V |
| 3) Shopping Centers   - Shopping Mall or Arcade - Strip Mall - Open-air Market | No/Yes  No/Yes  No/Yes |  | N/A^a^  N/A^a^  N/A^a^ | 100  100  100 |  | N/A^a^  N/A^a^  N/A^a^ | 98.5  100  98.5 |  | N/A^a^  N/A^a^  N/A^a^ | 100  100  100 | -  -  - |
| ***Streetscape*** | | | | | | | | | | |  |
| 1) Number of public transit stops |  | 0.98 |  | 96.9 | 0.82 |  | 63.1 | 0.87*** |  |  | V |
| 2) What is available at the first transit stop?   - Bus - BRT - Train - Subway - Tram/Streetcar - Bench - Covered Shelter - Timetable/Time | No/Yes  No/Yes  No/Yes  No/Yes  No/Yes  No/Yes  No/Yes  No/Yes |  | 0.80  N/A^a^  N/A^a^  N/A^a^  0.92  0.88  0.88  0.48 | 94.1  97.1  100  100  97.1  94.1  94.1  94.1 |  | N/A^a^  N/A^a^  N/A^a^  N/A^a^  0.75  0.42  0.47  N/A^a^ | 85.3  100  100  100  91.2  70.6  73.5  85.3 |  | N/A^a^  N/A^a^  N/A^a^  N/A^a^  0.91  0.60  0.66  N/A^a^ | 80.0  96.7  100  100  96.7  80.0  83.3  90.0 | V  -  -  -  V  V  V  V |
| 3) Are there informal places to catch transit? | No/Yes |  | N/A^a^ | 100 |  | N/A^a^ | 100 |  | N/A^a^ | 100 | - |
| 4) What other street characteristics are present?  a. Traffic calming (signs, circles, speed tables, speed humps, curb extension)  b. Roll-over curbs |  | 0.52  0.85 |  | 35.4  84.6 | 0.44  0.49 |  | 38.5  36.9 | 0.46***  0.32* |  |  | V  V |
| 5) Presence of street amenities   - Trash bins (public) - Benches or other places to sit - Bicycle racks - Secure bicycle access lockers or compounds - Bicycle docking stations - Kiosks or information booths - Hawkers/shops/carts | No/Yes  No/Yes  No/Yes  No/Yes  No/Yes  No/Yes  No/Yes |  | 0.84  0.72  0.74  N/A^a^  N/A^a^  N/A^a^  N/A^a^ | 92.3  86.2  87.7  100  100  100  100 |  | 0.63  0.50  0.72  N/A^a^  N/A^a^  N/A^a^  N/A^a^ | 81.5  73.8  86.2  100  100  76.9  98.5 |  | 0.72  0.54  0.71  N/A^a^  N/A^a^  N/A^a^  N/A^a^ | 86.2  76.9  86.2  100  100  100  100 | V  V  V  -  -  -  - |
| ***Aesthetics and Social*** | | | | | | | | | | |  |
| 1) Do you observe pleasant hardscape features, such as fountains, sculptures, or art (public or private)? | No/Yes |  | 0.51 | 92.3 |  | **0.23** | **70.8** |  | **0.24** | **80.0** | V |
| 2) Do you observe any natural bodies of water? | No/Yes |  | 0.91 | 96.9 |  | 0.75 | 90.8 |  | 0.74 | 90.8 | V |
| 3) Do you observe softscape features such as gardens or landscaping (e.g. designated viewpoints, retaining walls, bark, ponds)? | No/Yes |  | 0.50 | 76.9 |  | **0.36** | **73.8** |  | **0.18** | **56.9** | On-site |
| 4) Are the buildings well maintained? | 0%/ 1-49%/ 50-99%/ 100% |  | **0.32** | **66.2** |  | **0.00** | **41.5** |  | **0.25** | **61.5** | ? |
| 5) Is landscaping well maintained? | 0%/ 1-49%/ 50-99%/ 100% |  | **0.13** | **70.8** |  | **0.04** | **38.5** |  | **0.17** | **60.0** | ? |
| 6) Is graffiti/tagging (not murals) present? | No/Yes |  | **0.27** | **87.7** |  | 0.57 | 83.1 |  | **0.39** | **83.1** | V |
| 7) Is noticeable/excessive litter present? | No/Yes |  | 0.66 | 84.6 |  | **0.28** | **66.2** |  | **0.22** | **72.3** | On-site |
| 8) Is noticeable/excessive dog fouling present? | No/Yes |  | 0.45 | 90.8 |  | **-0.02** | **96.9** |  | **-0.03** | **87.7** | V |
| 9) Rate the extent of graffiti, litter and dog fouling. | None/ A little (present)/ Some (very noticeable)/ A lot (overwhelming) |  | 0.68 | 83.1 |  | 0.42 | 64.6 |  | **0.30** | **64.6** | On-site |
| 10) Presence of anyone walking? | No/ Yes |  | 0.45 | 78.5 |  | 0.87 | 95.4 |  | 0.57 | 81.5 | V |
| 11) Is there a highway (street which is 45mph+ or 5+ traffic lanes wide) nearby? | No/Yes |  | 0.48 | 93.8 |  | 0.57 | 93.8 |  | 0.57 | 93.8 | V |
| ***SEGMENT (n=220)*** | | | | | | | | | | |  |
| 1) How many traffic lanes are present (include traffic and turn lanes; choose most predominant)? |  | 0.83 |  | 91.8 | 0.89 |  | 95.4 | 0.81*** |  |  | V |
| 2) Is parking allowed on the segment? | None/ 1-25%/ 26-50%/ 51-75%/ 76-100% |  | 0.69 | 81.5 |  | 0.55 | 72.6 |  | 0.62 | 76.9 | V |
| 3) Is a continuous sidewalk present? | Yes, sidewalk is continuous/ No, sidewalk is not continuous/ No, no sidewalk |  | 0.97 | 98.6 |  | 0.46 | 80.0 |  | 0.82 | 91.2 | V |
| 4) What is the width of the majority of the sidewalk? | <3ft(1m)/ 3-5ft(1-1.5m)/ >5ft(1.5m)/ No sidewalk |  | 0.79 | 89.3 |  | **0.37** | **58.1** |  | **0.38** | **57.2** | On-site |
| 5) Is a buffer present? | No/ Yes/ Not applicable (no sidewalk) |  | 0.89 | 94.4 |  | 0.45 | 75.3 |  | 0.66 | 81.4 | V |
| 6) Are there poorly maintained sections of the sidewalk that constitute major trip hazards? (e.g. heaves, misalignment, cracks, overgrowth) | None/ One/ A few/ Many/ No sidewalk |  | 0.58 | 73.0 |  | 0.59 | 76.3 |  | 0.47 | 66.5 | V |
| 7) Are there hawkers or shops on the sidewalk or pedestrian street/zone? | None/ One/ A few/ Many/ No sidewalk-pedestrian zone |  | 0.95 | 98.2 |  | **0.04** | **80.3** |  | 0.78 | 92.2 | V |
| 8) Are there signs, bus shelters, kiosks and street furniture obstructing the sidewalk or pedestrian street/zone? | None/ One/ A few/ Many/ No sidewalk-pedestrian zone |  | 0.83 | 92.2 |  | 0.41 | 60.6 |  | 0.49 | 72.0 | V |
| 9) Are there cars blocking the sidewalk or pedestrian street/zone? | None/ One/ A few/ Many/ No sidewalk-pedestrian zone |  | 0.90 | 95.9 |  | **0.32** | **79.4** |  | 0.74 | 89.4 | V |
| 10) Is there an informal path (shortcut) which connects to something else? | No/ Yes |  | 0.77 | 95.9 |  | 0.84 | 97.2 |  | 0.58 | 92.7 | V |
| 11) What is the slope of the majority of the segment? | Flat or gentle/ Moderate/ Steep |  | **0.39** | **98.6** |  | **-0.01** | **99.1** |  | **-0.01** | **98.2** | V |
| 12) How many trees exist within 5 feet (1.5m) of either side of the sidewalk/pathway/other place to walk (can be in buffer or setback; also count trees that are more than 5 feet (1.5m) away if they provide shade)? | 0 or 1/ 2-5/ 6-10/ 11-20/ 21+/ Not applicable |  | 0.76 | 83.2 |  | 0.67 | 74.8 |  | 0.68 | 76.7 | V |
| 13) What percentage of the length of the sidewalk/walkway is covered by trees? | 1-25%/ 25-50%/ 51-75%/ 76-100%/ No coverage/ Not applicable |  | 0.61 | 80.9 |  | 0.63 | 75.1 |  | 0.52 | 73.1 | V |
| 14) What percentage of the length of the sidewalk/walkway is covered by awnings or other overhead coverage? | 1-25%/ 25-50%/ 51-75%/ 76-100%/ No coverage/ Not applicable |  | 0.87 | 95.5 |  | 0.55 | 76.1 |  | 0.47 | 73.1 | V |
| 15) What is the smallest building setback from the sidewalk/walkway? | No building/ 0ft/ 1-10ft(3m)/ 10-20ft(3-6m)/ 21-50ft(6-15m)/ 51-100ft(15-30m)/ >100ft(>30m) |  | 0.76 | 85.0 |  | 0.54 | 70.6 |  | 0.61 | 74.4 | V |
| 16) What is the largest building setback from the sidewalk/walkway? | No building/ 0ft/ 1-10ft(3m)/ 10-20ft(3-6m)/ 21-50ft(6-15m)/ 51-100ft(15-30m)/ >100ft(>30m) |  | 0.71 | 77.7 |  | 0.48 | 58.3 |  | 0.50 | 60.7 | V |
| 17) What is the shortest building height? (Count both sides of the street) | No building/ 1-3 stories/ 4-6 stories/ 7-12 stories/ 13-20 stories/ 21+ stories |  | 0.74 | 95.0 |  | 0.67 | 94.0 |  | 0.53 | 90.4 | V |
| 18) What is the tallest building height? (Count both sides of the street) | No building/ 1-3 stories/ 4-6 stories/ 7-12 stories/ 13-20 stories/ 21+ stories |  | 0.83 | 90.5 |  | 0.58 | 78.0 |  | 0.48 | 70.3 | V |
| 19) How many properties are protected by gates, walls or tall fences (6ft/2m or over)? | None/ 1-25%/ 26-50%/ 51-75%/ 76-100% |  | **0.23** | **89.5** |  | 0.42 | 77.5 |  | **0.22** | **78.1** | V |
| 20) How many driveways are there? Do not count alleys. | None/ 1-2/ 3-5/ 6+ |  | 0.74 | 81.4 |  | 0.67 | 77.1 |  | 0.66 | 75.3 | V |
| 21) Estimate the proportion of street segment that has ground floor or street-level windows within 40ft/12m of sidewalk/walkway (or street if no sidewalk/walkway) | 1-25%/ 26-50%/ 51-75%/ 76-100%/ No windows |  | 0.48 | 66.8 |  | 0.81 | 88.1 |  | 0.57 | 71.7 | V |
| 22) Is there a mid-segment crossing? | No/ Yes |  | 0.85 | 95.0 |  | **0.33** | **90.8** |  | **0.36** | **84.0** | V |
| 23) If yes, is it a pedestrian bridge/overpass or a tunnel? | No/ Yes/ Not applicable |  | 0.85 | 95.0 |  | **0.30** | **89.9** |  | **0.34** | **83.1** | V |
| 24) Is there a covered or air conditioned place to walk along the street or connecting buildings (not a mall)? | No/ Yes |  | N/A^a^ | 100 |  | N/A^a^ | 98.6 |  | N/A^a^ | 100 | - |
| 25) Is there a bicycle lane or zone? Select one. | Yes, on the sidewalk/ Yes, separated form traffic by a marked line/ Yes, separated from traffic by a raised curb/ Yes, separated from traffic by a buffer (plantings, parked cars, fencing, etc)/ No |  | 0.89 | 97.3 |  | 0.83 | 95.4 |  | 0.86 | 96.3 | V |
| 26) What is the quality of the bicycle lane or zone? | Poor/ Fair/ Excellent/ Not applicable (no bike lane or zone) |  | 0.70 | 92.7 |  | 0.95 | 98.6 |  | 0.75 | 93.6 | V |
| 27) Are there signs or sharrows indicating bicycle use? | No/ Yes |  | 0.51 | 95.9 |  | **0.38** | **88.5** |  | **0.31** | **91.3** | V |
| 28) How many high (car) street lights are installed? | None/ Some/ Ample |  | 0.61 | 75.0 |  | 0.55 | 72.5 |  | 0.50 | 69.4 | V |
| 29) How many low (pedestrian) street lights are installed? | None/ Some/ Ample |  | 0.75 | 97.3 |  | 0.65 | 95.9 |  | 0.64 | 96.3 | V |
| ***CROSSING (n=124)*** | | | | | | | | | | |  |
| 1) Intersection control   - Yield signs - Stop signs - Traffic signal - Traffic circle | No/Yes  No/Yes  No/Yes  No/Yes |  | 0.75  **-0.01**  0.83  N/A^a^ | 88.6  **98.4**  98.4  100 |  | 0.90  0.66  1.00  N/A^a^ | 95.9  99.2  100  100 |  | 0.56  **-0.01**  0.85  N/A^a^ | 80.5  **98.4**  98.4  100 | V  V  V  - |
| 2) Does this crossing take place on an overpass, underpass or bridge? | No/Yes |  | N/A^a^ | 100 |  | N/A^a^ | 99.2 |  | N/A^a^ | 100 | - |
| 3) Signalization   - Pedestrian walk signals - Push buttons - Countdown signal - Bicycle signal | No/Yes  No/Yes  No/Yes  No/Yes |  | 1.00  1.00  N/A^a^  N/A^a^ | 100  100  100  99.2 |  | 0.91  1.00  1.00  N/A^a^ | 99.2  100  100  99.2 |  | 1.00  1.00  N/A^a^  N/A^a^ | 100  100  99.2  99.2 | V  V  V  - |
| 4a) Pre-crossing curb | Ramp lines up with crossing/ Ramp does not line up with crossing/ No ramp |  | 0.75 | 89.4 |  | 0.69 | 84.4 |  | 0.64 | 84.6 | V |
| 4b) Post-crossing curb | Ramp lines up with crossing/ Ramp does not line up with crossing/ No ramp |  | 0.71 | 88.6 |  | 0.77 | 88.5 |  | 0.64 | 85.4 | V |
| 5) Is tactile paving provided at curbs? | Yes, at one curb/ Yes, both curbs/ No |  | 0.88 | 98.4 |  | 0.94 | 99.2 |  | 0.76 | 96.8 | V |
| 6) Are crossing aids (e.g. flags) present? | No/ Yes |  | N/A^a^ | 100 |  | N/A^a^ | 100 |  | N/A^a^ | 100 | - |
| 7) Crosswalk treatment   - Marked crosswalk - High-visibility striping - Different material than road - Curb extension - Raised crosswalk | No/ Yes  No/ Yes  No/ Yes  No/ Yes  No/ Yes |  | N/A^a^  1.00  **0.34**  N/A^a^  N/A^a^ | 100  100  **94.3**  100  99.2 |  | N/A^a^  0.94  0.62  0.49  **0.38** | 99.2  97.5  91.8  98.4  **95.1** |  | N/A^a^  0.96  **0.19**  N/A^a^  N/A^a^ | 99.2  98.4  **88.6**  97.6  94.3 | -  V  V  V  V |
| 8) Is a protected refuge island present? | No/ Yes |  | 1.00 | 100 |  | 0.43 | 97.5 |  | 0.67 | 98.4 | V |
| 9) Is there poor visibility at the corners, around roundabouts, or from parked cars? | No/ Yes |  | **0.38** | **78.0** |  | **0.27** | **76.2** |  | **-0.02** | **61.8** | On-site |
| 10) Distance of crossing leg, including all traffic lanes |  | 0.90 |  | 95.9 | 0.78 |  | 89.3 | 0.79*** |  |  | V |
| 11) Is a waiting area (bike box) provided for cyclists who stop at the crossing? | No/ Yes |  | 0.80 | 99.2 |  | 0.66 | 99.2 |  | 0.66 | 99.2 | V |
| 12) Does a bike lane or path cross the crossing? | No/ Yes |  | 0.90 | 99.2 |  | 0.76 | 97.5 |  | 0.65 | 96.7 | V |
| ***CUL-DE-SAC/DEAD END (n=6)*** | | | | | | | | | | |  |
| 1) How close is the cul-de-sac or dead-end opening to the participants’ home? | On the cul-de-sac  Adjacent to the cul-de-sac (one or two homes/houses removed from cul-de-sac opening)  Non-adjacent, but less than 200ft(60m) away  More than 200ft(60m) away |  | 0.76 | 83.3 |  | 0.76 | 83.3 |  | 0.76 | 83.3 | V |
| 2) What amenities exists at the opening to or along the cul-de-sac or dead-end portion of the street?   - Basketball hoops - Skateboard features (e.g. ramps) - Soccer goals - Outdoor fitness equipment |  |  | N/A^a^  N/A^a^  N/A^a^  N/A^a^ | 100  100  83.3  100 |  | N/A^a^  N/A^a^  N/A^a^  N/A^a^ | 100  100  100  100 |  | N/A^a^  N/A^a^  N/A^a^  N/A^a^ | 100  100  83.3  100 | -  -  -  - |
| 3) Can most of the cul-de-sac or dead-end area be seen from the participant’s home (using the most optimal viewpoint form the home, including higher story windows)? |  |  | 0.67 | 83.3 |  | 0.67 | 83.3 |  | 1.00 | 100 | V |
| ^a^ Unable to be calculated as at least one variable is constant // *** p<0.001 **p<0.01 *p<0.1  ICC: intraclass correlation coefficient; r: Pearson correlation coefficient  **Bold: Fair, poor or no agreement (ICC/kappa: 0.00 – 0.40) and high percentage agreement (> 70%)**  **Bold underlined: Fair, poor or no agreement ((ICC/kappa: 0.00 – 0.40) and low percentage agreement (< 70%) or low Pearson correlations (≤0.30)**  Interpretation ‘usability’: V (reliable item that can be observed on-site or by Google Street View in Belgium); on-site (item that should be observed on-site in Belgium); ? (unreliable item that might need adjustments); - (item that are rare or nonexistent in Belgium and should be rated in other Belgian regions and other countries) | | | | | | | | | | | |
